# Surface guided radiotherapy (SGRT) improves breast cancer patient setup accuracy

**DOI:** 10.1002/acm2.12700

**Published:** 2019-09-03

**Authors:** Malin Kügele, Annika Mannerberg, Susanne Nørring Bekke, Sara Alkner, Lovisa Berg, Faisal Mahmood, Charlotte Thornberg, Anneli Edvardsson, Sven Å. J. Bäck, Claus F. Behrens, Sofie Ceberg

**Affiliations:** ^1^ Department of Hematology, Oncology and Radiation Physics Skåne University Hospital Lund Sweden; ^2^ Medical Radiation Physics, Department of Clinical Sciences Lund University Lund Sweden; ^3^ Radiotherapy Research Unit, Department of Oncology Herlev Hospital, University of Copenhagen Copenhagen Denmark; ^4^ Department of Clinical Sciences, Division of Oncology and Pathology Lund University Lund Sweden; ^5^ Department of Oncology Odense University Hospital Odense C Denmark

**Keywords:** interfraction motion, optical surface scanning, patient positioning, surface guided radiotherapy

## Abstract

**Purpose:**

The purpose of the study was to investigate if surface guided radiotherapy (SGRT) can decrease setup deviations for tangential and locoregional breast cancer patients compared to conventional laser‐based setup (LBS).

**Materials and Methods:**

Both tangential (63 patients) and locoregional (76 patients) breast cancer patients were enrolled in this study. For LBS, the patients were positioned by aligning skin markers to the room lasers. For the surface based setup (SBS), an optical surface scanning system was used for daily setup using both single and three camera systems. To compare the two setup methods, the patient position was evaluated using verification imaging (field images or orthogonal images).

**Results:**

For both tangential and locoregional treatments, SBS decreased the setup deviation significantly compared to LBS (*P* < 0.01). For patients receiving tangential treatment, 95% of the treatment sessions were within the clinical tolerance of ≤ 4 mm in any direction (lateral, longitudinal or vertical) using SBS, compared to 84% for LBS. Corresponding values for patients receiving locoregional treatment were 70% and 54% for SBS and LBS, respectively. No significant difference was observed comparing the setup result using a single camera system or a three camera system.

**Conclusions:**

Conventional laser‐based setup can with advantage be replaced by surface based setup. Daily SGRT improves patient setup without additional imaging dose to breast cancer patients regardless if a single or three camera system was used.

## 1 INTRODUCTION

**Figure 1:**
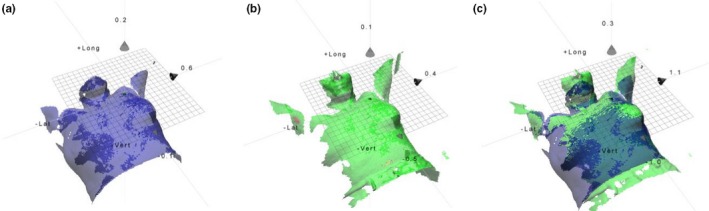
(a) Reference surface (blue color) with the planned isocenter from the treatment planning system. (b) The live patient surface (green color) captured by a single camera Catalyst^TM^ system. (c) The reference and live surface are matched with a deformable algorithm and a couch shift in 6° of freedom is calculated to shift the live surface into the correct position with respect to the isocenter.

Breast conserving surgery can remove macroscopic disease for early stage breast cancer.^1^ After surgery some microscopic tumor foci may remain, and if not treated with radiotherapy this can lead to locoregional recurrence and/or life‐threatening distant metastases.^1^ Early Breast Cancer Trialists' Group performed a meta‐analysis of individual data for 10 801 women from 17 randomized trials and showed that the 10‐yr risk for any first recurrence was 35% for women allocated to breast conserving surgery only, and 19% for women allocated to breast conserving surgery and radiotherapy.^1^ The absolute risk reduction was 16%. For every four recurrence avoided by radiotherapy, one breast cancer death can be avoided.^1^ There is no effective method to find microscopic disease after breast conserving surgery and therefor radiotherapy is still considered to be important for the cure of breast cancer. Radiotherapy for breast cancer treatment uses a three‐dimensional computed tomography (3DCT)‐based treatment planning which enables a high local selectivity for the dose distribution; the target tissue is irradiated while the normal tissue is spared. The treatment planning system (TPS) ensures a high accuracy in the dose deposition which requires high accuracy in daily patient setup. Breast cancer patients have a long expected survival and it is of importance to reduce interfractional setup errors to avoid excessive irradiation that can cause toxicity in normal healthy tissue. The organs at risk (OAR) are primarily the lung and the heart. Hence, complications such as radiation pneumonitis and cardiac mortality have been shown to positively correlate with the volume irradiated.^2^, ^3^ Setup verification imaging strategies, generally classified as either online or offline, are used to ensure that systematic and randomized setup deviations are minimized throughout treatment. The drawback is that both strategies are associated with a risk for second malignancies due to imaging dose.^4^ The online strategy implies daily imaging before treatment with a preset threshold for deviations. Laaksomaa et al., recommended daily online image guidance due to large random interfractional variation in patient posture.^5^ This strategy is time‐consuming and contributes imaging dose to the patient throughout treatment. Having in mind the increased radiation dose due to imaging, the ALARA principle and the fact that the survival of breast cancer patients is expected to be long, an accurate nondose‐contributing setup system is warranted. The offline strategy requires frequent imaging in the beginning of the treatment course. The result is statistically analyzed for the systematic and random components of the deviation in the patient position. The systematic deviation is compensated for by a couch shift for the following treatment sessions.^6^ The random deviation is mainly due to the inaccuracy in laser aligned setup, which is commonly used for daily setup. The patients are aligned according to landmarks on the skin and room lasers.^6^ An alternative approach is to use surface guided radiotherapy (SGRT), which uses a three‐dimensional (3D) model of the skin surface for positioning and monitoring. The optical surface scanning (OSS) system compares a 3D model of the patient’s external surface extracted from the TPS with a live scan of the surface while the patient is positioned on the treatment couch (Fig. 1). Surface based setup (SBS) increases the patient setup information compared to laser‐based setup (LBS), by using the entire patient skin surface instead of only three skin marks. Several OSS systems have shown a high correlation with verification imaging results.^7^, ^8^, ^9^ Also, Chang et al. have in a study with 23 patients shown that SGRT has a high correlation to the lumpectomy cavity defined by surgical clips for breast cancer patients receiving accelerated partial breast irradiation.^10^ The OSS system Catalyst^TM^ (C‐rad Positioning AB, Uppsala, Sweden) has been evaluated in this study. This OSS system is unique because it uses a deformable algorithm to calculate the isocenter position. The principle behind the deformable registration in depth scans is described by Hao Li et al.^11^ Recently published results showed that patient setup using the deformable algorithm of the Catalyst^TM^ system was superior to LBS for breasts with nodal involvement in TomoTherapy (Accuray, Sunnyvale, CA).^12^ The work carried out by Crop et al. used mass‐weighted PTV location for patient setup, specially designed for the TomoTherapy environment. Similar results were observed at a linear accelerator by Stanley et al. using the Catalyst^TM^ for breast cancer patient positioning.^13^ However, comparison between tangential and locoregional treatments and single *vs.* three camera systems has to our knowledge not been investigated.

Tangential and locoregional treatments, and also, single and three camera systems result in different surface coverage which motivates an investigation of how the setup accuracy is affected. The aim of this study was to retrospectively compare LBS with SBS using the OSS system Catalyst^TM^ for both tangential and locoregional breast cancer patients using single and three camera systems.

## 2 MATERIALS AND METHODS

### 2.1 Ethical consideration and consent

The use of the radiotherapy database for retrospective research has been approved by the Regional Ethical Review Board in Lund (No. 2013/742).

### 2.2 Patient selection

A total of 139 patients were enrolled in this study, 63 patients received tangential treatment after breast conserving surgery and 76 with locally advanced breast cancer patients received locoregional treatment after mastectomy or breast conserving surgery. Both left‐ and right‐sided breast cancers were included, however, patients treated in deep inspiration breath hold were excluded in this study. The median age was 62 yr (range: 34–83 yr) and 64 yr (range: 33–87 yr) for the breast cancer patients receiving tangential and locoregional treatment, respectively.

### 2.3 Computed tomography and patient immobilization

All patients underwent CT using a Siemens Somatom definition AS plus (Siemens Medical Solutions, Erlangen, Germany) for treatment planning. In the TPS (Eclipse version 10.0.28 and 13.6.23, Varian medical systems; CA Varian), the surface structure set (BODY), treatment fields and isocenter position were exported to the Catalyst^TM^ in the industry standard DICOM format. The patients were treated in supine position on a breast board (Posiboard^TM^‐2 Breastboard, CIVCO Medical Solutions) with their arms raised over the head and positioned on an arm support. For tangential and locoregional treatment, a breastboard pitch of 7.5° and 0° was used as standard, respectively. An immobilization wedge was placed under the patients’ knees for support. One patient receiving locoregional treatment was positioned in a WingStep^TM^ (Elekta AB, Stockholm, Sweden) and body vacuum bag with the contralateral arm by the side of her body.

### 2.4 Treatment plans

The treatment prescription was 50 Gy in 25 fractions or 42.6 Gy in 15 fractions, normalized to the PTV mean or median dose. In the TPS 3D conformal treatment plans were created for all patients. For the tangential treatments, two opposing 6 MV tangential fields to cover the breast tissue was used. Also, a supplementary field and/or wedges were used for dose homogenization purposes. The isocenter position was placed central in the breast tissue. For the locoregional treatments, opposing tangential fields were used to cover the location of the breast tissue. To complement the tangential fields in order to achieve homogeneous dose a various number of supplementary fields of 6 or 10 MV were used. The number of fields used depended on target size and patient anatomy. The locoregional axillary lymph nodes were covered with a 6 MV anterior‐posterior (AP) field and a 10 MV posterior‐anterior (PA) field. Also, a supplementary 10 MV PA field was used while shielding of the lung tissue. The total number of fields used for the locoregional treatments ranged between six and nine. For mastectomy patients, a 0.5‐cm thick and 6‐cm wide bolus (Superflab, Mick Radio‐Nuclear Instruments, Inc. An Eckert & Ziegler BEBIG Company) was placed over the operation scar. For locoregional treatment, the treatment isocenter was positioned in the junction between the tangential and AP‐PA fields.

### 2.5 Surface guided radiotherapy with a deformable algorithm for isocenter calculation

The OSS systems were ceiling‐mounted in nine treatment rooms, as either a single camera or a three camera configuration. The three camera configuration provides a 360° surface coverage of the patient, due to a 120° installation angle between the systems. For the single camera configuration, only one system is scanning the patient, thus, the surface coverage becomes degraded. OSS systems at six Varian TrueBeam (Varian Medical Systems, Palo Alto, USA) and three ELEKTA Synergy (Elekta AB, Stockholm, Sweden) equipped with verification imaging systems were used. The single camera Catalyst^TM^ configuration was used for setup of all the tangential treatments. For locoregional treatments, both the single and three camera Catalyst^TM^ configuration were used for patient setup.

The Catalyst^TM^ system consists of a high‐power LED projector, which projects a near‐visible violet light (λ = 405 nm) for surface reconstruction purposes and a green (λ = 528 nm) and red (λ = 624 nm) projection light for live feedback of the patient posture.^14^ The near‐visible violet light is projected as sequenced lines onto the object to be scanned. The irregularity of the object scanned disperses the sequenced lines, which is detected by a charged‐coupled device (CCD) camera. Due to fixed geometry between the projector and the CCD, the principle of optical triangulation can be used to reconstruct a 3D surface of the object scanned.^15^ Patient setup with the Catalyst^TM^ was carried out in two steps; (a) patient posture correction using surface matching and (b) isocenter position adjustment using a deformable algorithm. To correct for patient posture, the OSS system matches the reference surface with the live surface within a determined scanning volume and creates a distance map between the two surfaces. If the two surfaces differ from a preset surface tolerance, the system creates a color map that is back‐projected onto the patient’s skin. The therapists manually correct the patient posture, and the color map turns transparent once the two surfaces are within the surface tolerance. Based on how well the reference and live surface match the system carries out a depth calculation of the isocenter position using a deformable algorithm (Fig. 1).^16^ Thus, the calculated isocenter shift depends on the daily patient setup. For the isocenter calculation, the full patient surface coverage of the thorax was used, however, surface close to isocenter is weighted higher in the calculation than distant surface. Also, anatomical deformations that can occur during the course of radiotherapy were automatically handled by the algorithm. For each patient, the scanning parameters were adjusted individually in the Catalyst^TM^ software to obtain optimal image quality, minimize camera shadowing, and over or under exposed images.

### 2.6 Patient setup protocol

At the treatment machine, all patients were initially positioned by laser alignment to a 3‐point based tattoo setup.

For the patients positioned using LBS, the calculated shift from the reference point to the isocenter position was manually carried out the first treatment session and the isocenter position was drawn onto the patient’s skin using a marker pen. Verification images were acquired, according to a No Action Level (NAL) offline strategy.^17^ The systematic deviation was estimated after the first three treatment sessions and the setup was corrected for the remaining treatment sessions. To carry out a fair comparison between the LBS and SBS setup strategy, the setup data from the three first treatment session for the patients positioned using LBS were excluded.

**Figure 2:**
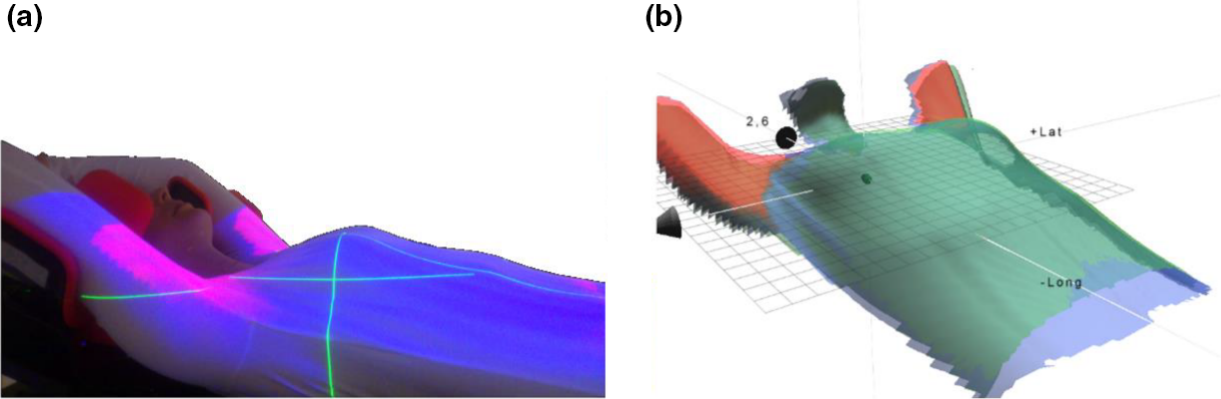
(a) Color map projected onto the patient’s skin for live visual guidance of posture errors in the patient setup. (b) The color map is also shown in the software inside the treatment and control room.

For the patients positioned using SBS, the couch was shifted to the treatment position and the correction for posture was performed using the color map with a tolerance of 5 mm (Fig. 2). The effect of the free breathing motion was minimized by using a floating mean value calculation over 4 s for the live image. Once the posture was within the surface tolerance, the therapists manually shifted the couch to correct for the isocenter deviation. The patient setup result was saved by the therapists inside the treatment room and a residual isocenter deviation ≤2 mm, and rotations ≤ 3° were accepted in this study.

**Figure 3:**
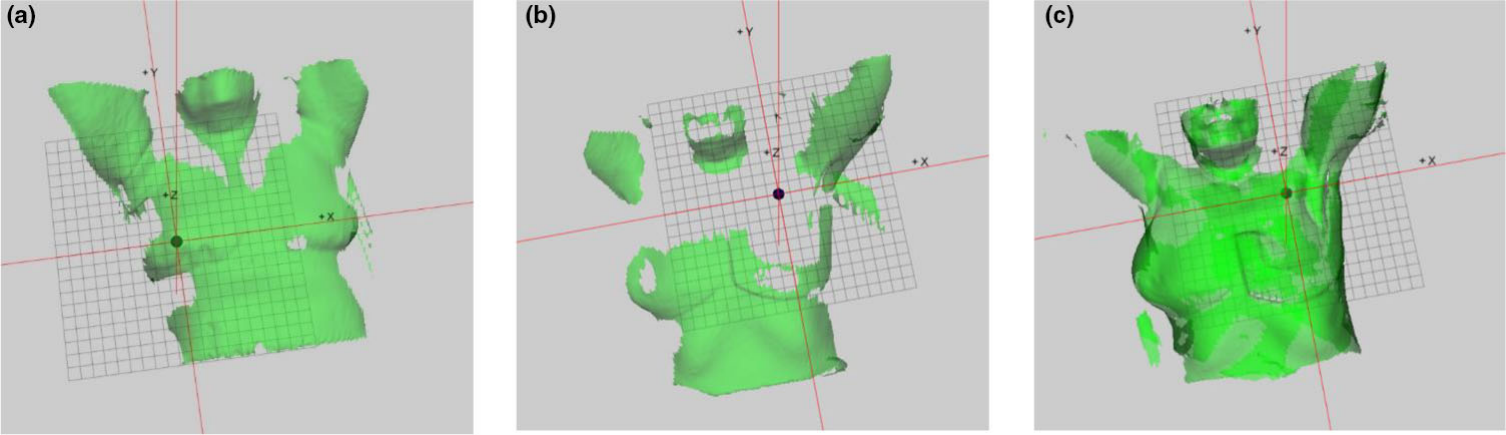
(a) Surface of a patient receiving tangential breast cancer treatment, positioned with a single camera system. The breast board pitch of 7.5° enhances the patient surface coverage. The isocenter is located in the breast tissue. (b) Surface of a patient receiving locoregional breast cancer treatment at a single camera Catalyst^TM^ system. Nonoptimal camera settings in combination with a 0° pitch of the breast board cause shadowing and the bolus occludes the signal. The loss of patient surface is above the isocenter. (c) Surface of a patient receiving locoregional breast cancer treatment at a three camera Catalyst^TM^ system, with optimal camera settings. Full surface coverage of the patient, including the bolus, is observed.

For both the tangential and locoregional treatments, each patient was positioned using either SBS or LBS and the position was verified using onboard imaging at the linac. Different patient anatomies were included for all four groups. The shifts in lateral (lat), longitudinal (lng) and vertical (vrt) direction, respectively, and the total vector offset ($v=\sqrt{{\mathrm{lat}}^{2}+{\mathrm{lng}}^{2}+{\mathrm{vrt}}^{2}}$), were evaluated. For SBS, the variation in patient anatomies caused more or less camera shadowing in the live image (Fig. 3).

#### 2.6.1 Tangential treatment

A total of 65 patients, 28 patients with LBS and 37 patients with SBS, were positioned using a single camera system. The two different setup techniques were verified with field images to enable comparison of the breast position in the treatment field. The anatomical landmarks used were the lung edge and the breast tissue. In total, 677 field images were evaluated. For comparison, a two‐sided Wilcoxon sum rank test was carried out for the vector offset and Students *t*‐test for two independent mean for the lat, lng, and vrt directions, with a statistical level of significance α = 0.01.

#### 2.6.2 Locoregional treatment

Three patient groups of totally 76 patients were enrolled in this study. For SBS, 43 patients were included; 22 patients positioned using a three camera system and 21 patients positioned using a single camera system. Nineteen of the patients had bolus over the operation scar, and one patient had a 1‐cm thick wet towel as a bolus. One patient was excluded due to that the OSS system was not used according to the study protocol. In the LBS group, 34 patients were enrolled. The patient setup was verified with orthogonal kilovolt (kV) or megavolt (MV) images. The anatomical landmarks used were the clavicular bone position, the lung edge, and sternum. In total, 632 verification images were evaluated. For comparison a two‐sided Wilcoxon sum rank test was carried out for the vector offset and Students t‐test for two independent mean for the translational directions with a statistical level of significance α = 0.01.

A two‐sided Wilcoxon sum rank test was carried out to investigate if there were any statistical significant difference between the single and three camera system with a significance level of 0.01.

## 3 RESULTS

### 3.1 Tangential treatment

**Figure 4:**
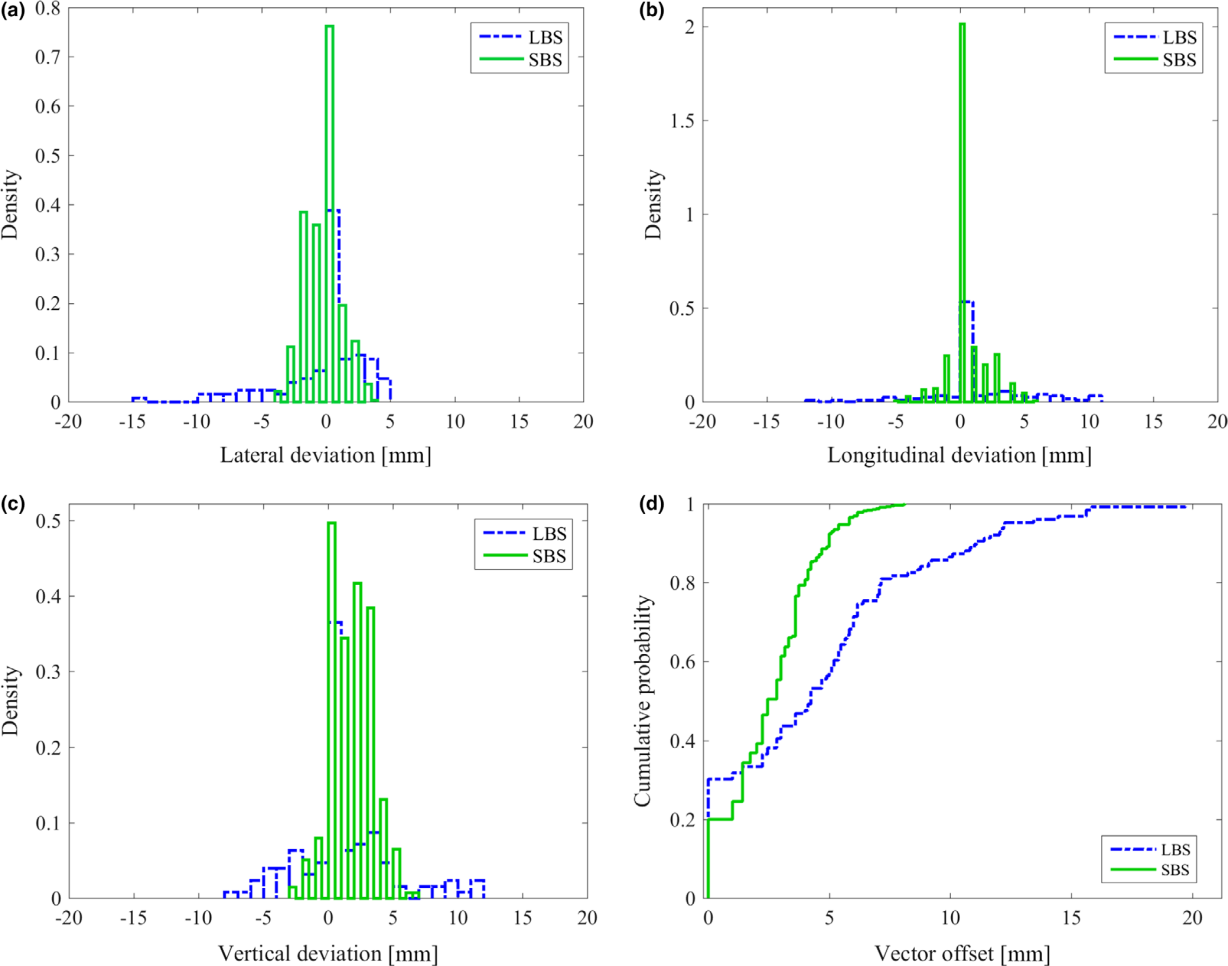
Setup deviation for breast cancer patients receiving tangential treatment positioned using laser‐based setup (LBS) and surface based setup (SBS). Histograms of the setup accuracy in (a) lateral, (b) longitudinal, and (c) vertical direction verified with field imaging. Reduced maximal deviations can be observed for SBS compared to LBS in all three translational directions. (d) The cumulative probability of the vector offset show a significantly improved patient setup for SBS compared to LBS (*P* < 0.01).

The median vector offset was 4.2 mm (range: 0–19.7 mm) for LBS and 2.4 mm (range: 0–8.1 mm) for SBS verified with field imaging (*P* < 0.01). For LBS and SBS, 84% and 95% of the treatment sessions were within the clinical tolerance of ≤4 mm in all the three directions (lat, lng, or vrt). The cumulative probability for positioning a patient within a spatial vector of 5.0 mm from isocenter was 57% for LBS and 89% for SBS (Fig. 4). For 90% of the setup cases, the spatial vector was within 11.0 mm for LBS and 5.0 mm for SBS. For LBS, the mean value (±1 SD) was −0.6 ± 3.3 mm, 0.8 ± 3.7 mm, 0.6 ± 3.7 mm in lat, lng, and vrt direction, respectively. For SBS, the mean value was −0.5 ± 1.4 mm, 0.4 ± 1.5 mm, 1.5 ± 1.7 mm in lat, lng, and vrt direction, respectively (Fig. 4). Significant difference was found in the vrt direction (*P* < 0.01).

### 3.2 Locoregional treatment

**Figure 5:**
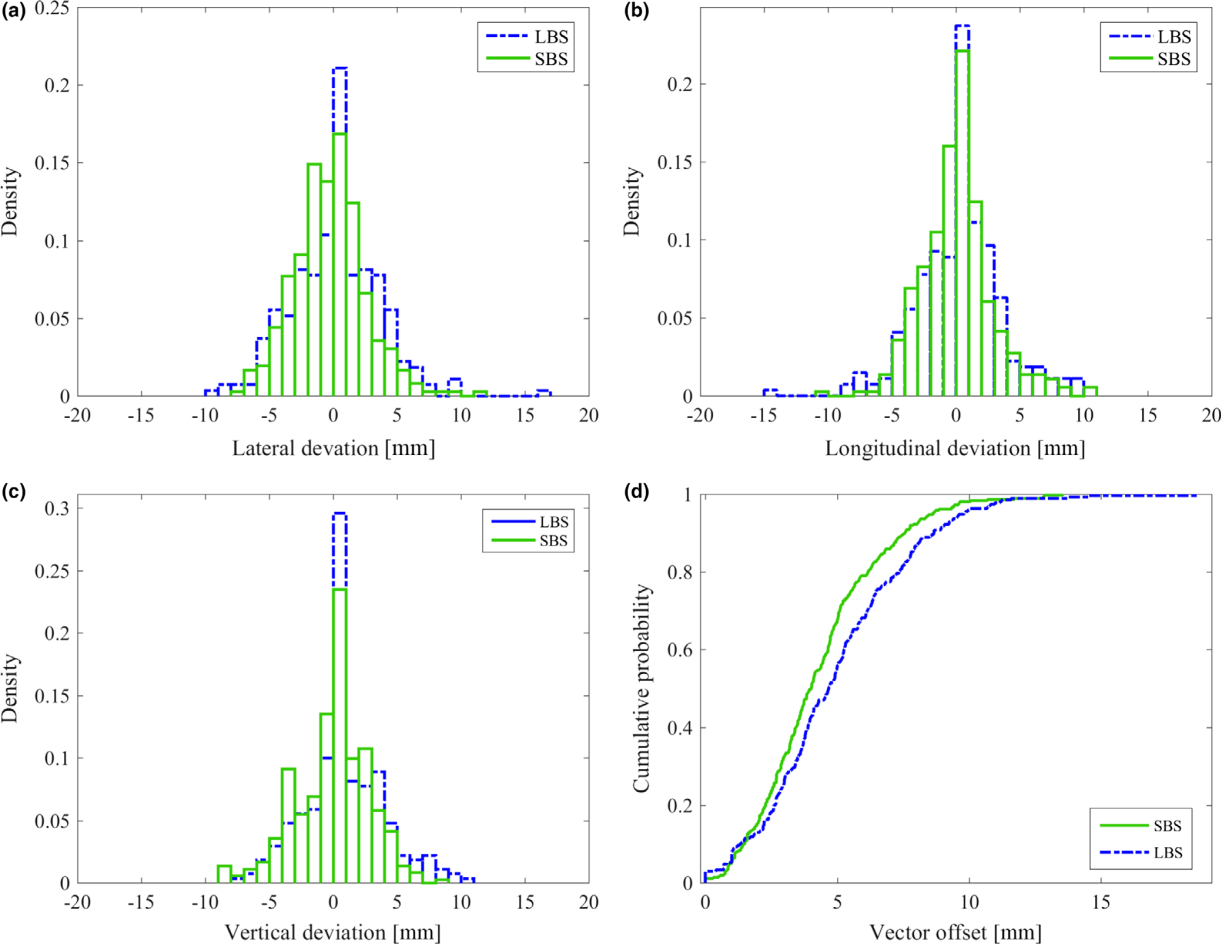
Setup deviation for breast cancer patients receiving locoregional treatment positioned using laser‐based setup (LBS) and surface based setup (SBS). Histograms of the setup accuracy in (a) lateral, (b) longitudinal, and (c) vertical direction verified with field imaging. (d) The cumulative probability of the vector offset shows a significantly improved patient setup for SBS compared to LBS (*P* < 0.01).

**Figure 6:**
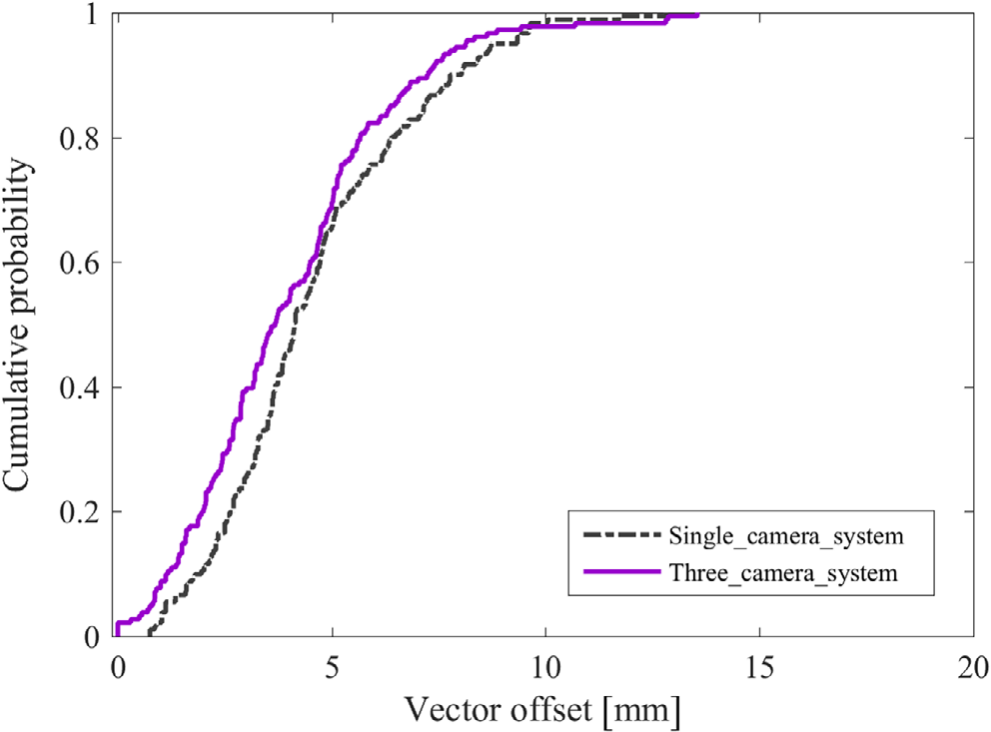
For the breast cancer patients receiving locoregional treatment, a total of 362 verification images were evaluated for patients positioned using SBS at a single camera Catalyst system (181 verification images) and a three camera Catalyst system (181 verification images). The cumulative probability of the vector offset shows a nonsignificant improved patient setup for three camera compared to single camera system (*P* = 0.02).

The median vector offset was 4.7 (0–18.7 mm) and 4.0 (range: 0−13.5 mm) for LBS and SBS, respectively (p < 0.01). For LBS, the mean value (±1 SD) was 0.1 ± 3.4 mm, 0.1 ± 3.3 mm, 0.7 ± 3.1 mm in lat, lng, and vrt directions, respectively. For SBS, the mean value (±1 SD) was −0.5 ± 2.8, −0.1 ± 2.8, −0.3 ± 2.9 mm in lat, lng, and vrt directions, respectively. The result was statistically significant for lat and vrt directions (*P* < 0.01). The cumulative probability for positioning a patient within a spatial vector of 5 mm from isocenter was 55% for LBS and 67% for SBS (Fig. 5). For 90% of the treatment sessions, the spatial vector was within 9.1 and 7.6 mm for LBS and SBS, respectively. For LBS and SBS, 54% and 70% of all treatment sessions were within the clinical tolerance of ≤4 mm in all three directions (lat, lng, vrt), respectively (Fig. 5). A small but not significant difference was observed (*P* = 0.02) for the vector offset, comparing the single camera system with the three camera system for SBS (Fig. 6).

## 4 DISCUSSION

For both the patient groups receiving tangential and locoregional breast cancer treatment, the patient setup was significantly improved using the Catalyst^TM^ system. For locoregional treatments, the clinical criteria (≤4 mm) were fulfilled for 16% more treatment sessions using SBS compared to LBS. The corresponding improvement for tangential treatments was 11%. This could potentially lead to a reduced amount of verification imaging in the clinic. Also, the standard setup deviation for patients receiving tangential treatment was approximately reduced by half. For a few treatment sessions, deviations up to 11 mm were observed for locoregional breast cancer patients using SBS, which also was observed by Stanley et al. using CBCT as verification imaging.^13^ In both LBS and SBS groups, large setup deviations were found when the patient’s arm was incorrectly positioned. For SBS, the therapists manually corrected the patient setup according to the criteria for posture, however, for a few treatments sessions, the criteria were not achievable due to shoulder stiffness after surgery.

The back‐projected color map had a great impact on correction of patient posture to the planned position, while LBS deficiencies were largely caused by the lack of information of the patient posture. Degraded image quality for SBS was observed for a few patients, due to nonoptimal camera settings. This was observed for individual patients, and also, for the surface covered with bolus. The camera exposure setting was optimized to scan the patient's skin color, and since the color of the bolus deviated from the patient skin color the camera got overexposed in this area. In the area of overexposure, the Catalyst^TM^ fails to reconstruct a surface, hence, information about the bolus position will be lost. Also, since the deformable algorithm values area above the isocenter in the calculation, an area where the bolus often is positioned, vital information is lost. For a single camera system and a locoregional patient with bolus, the surface that was covered was the lower parts of the thorax, arm, and chin. Since the arm and chin are not optimal anatomical structures to use for patient setup, this contributes to inaccuracy in this study. For the three camera system, better surface coverage over the treatment area was observed, contributing to a more accurate patient setup. The single camera system was installed in the ceiling by the foot end of the couch. The reconstructed surface depended on how much of the patient surface the camera was able to detect. For tangential treatments, a breastboard pitch of 7.5° was used which favored the Catalyst^TM^ camera, hence, the patient was tilted toward the camera. For patients receiving locoregional treatments, a breastboard pitch of 0° was used and in combination with a cranial isocenter, important surface above the isocenter was not covered using the single camera system. This loss of surface had a negative impact on the accuracy of the patient setup (Fig. 3). For 17 out of the 76 patients in this study, the mass center of the PTV was used instead of the isocenter for the setup calculation in the Catalyst^TM^ system. However, since the surface above the calculation point was lost, the setup accuracy was similar to using the calculated isocenter. For the three camera system, for locoregional treatment, and single camera for tangential treatment, the treatment site was well covered, which according to our results, as well as in the study of Chang et. al, leads to accurate positioning.^10^ In the time span between the in room SBS and the verification imaging during treatment, patient motion contributed to inaccuracy. For example, during one treatment session for one patient, an offset of −3, −6 and −3mm in lat, long and vrt direction, respectively, was observed for SBS. The Catalyst^TM^ log showed that the registered shifts were caused by patient motion between the setup and the verification imaging. Also, verification images (MV or kV) are snapshots of the patient position, while the OSS system was averaging the patient position over 4 s to reduce the effect of the breathing motion which also might contribute to uncertainty. Another source of error was patient rotation, which was observed to be larger than the 3° tolerance in the study protocol for individual treatment sessions using SBS. Rotations >2° has previously been reported by Guckenberger et al in 26% of patients with thoracic tumors, with a maximal rotational error of 8°.^18^ The authors could not observe any correlation between the rotational error and the magnitude of the translational error. Since three degree of freedom couches cannot compensate for rotations, an advantageous feature using SBS is the ability to manually correct for rotations inside the treatment room prior to verification imaging and/or treatment.

## 5 CONCLUSION

This study showed that surface based setup, using the Catalyst^TM^ system can replace the conventional laser‐based setup for tangential and locoregional breast cancer treatments, regardless if a single or a three camera system was used. Additional information of the patient posture was provided using surface based setup compared to laser‐based setup, which improved positioning. Daily surface guided radiotherapy for breast cancer patients can thus reduce time and dose associated with verification imaging.

## CONFLICT OF INTEREST

All authors approved the final manuscript, and declared that they have no potential conflict of interest to this work.
